# High-resolution dietary reconstruction of victims of the 79 CE Vesuvius eruption at Herculaneum by compound-specific isotope analysis

**DOI:** 10.1126/sciadv.abg5791

**Published:** 2021-08-25

**Authors:** Silvia Soncin, Helen M. Talbot, Ricardo Fernandes, Alison Harris, Matthew von Tersch, Harry K. Robson, Jan K. Bakker, Kristine K. Richter, Michelle Alexander, Steven Ellis, Gill Thompson, Valeria Amoretti, Massimo Osanna, Marina Caso, Francesco Sirano, Luciano Fattore, Andre C. Colonese, Peter Garnsey, Luca Bondioli, Oliver E. Craig

**Affiliations:** 1 BioArCh, Department of Archaeology, University of York, York, UK.; 2 Department of Archaeology, Max Planck Institute for the Science of Human History, Jena, Germany.; 3 School of Archaeology, University of Oxford, Oxford, UK.; 4 Faculty of Arts, Masaryk University, Brno, Czech Republic.; 5 Department of Archaeology and Classical Studies, Stockholm University, 10691 Stockholm, Sweden.; 6 ACASA, University of Amsterdam, Amsterdam, Netherlands.; 7 Department of Classics, University of Cincinnati, Cincinnati, OH 45221, USA.; 8 School of Archaeological and Forensic Sciences, University of Bradford, Bradford, UK.; 9 Parco Archeologico di Pompei, Naples, Italy.; 10 Parco Archeologico di Ercolano, Naples, Italy.; 11 Dipartimento di Biologia Ambientale, Sapienza Università di Roma, Rome, Italy.; 12 Department of Prehistory and Institute of Environmental Science and Technology (ICTA), Universitat Autònoma de Barcelona, Bellaterra, Spain.; 13 Faculty of History, University of Cambridge, Cambridge, UK.; 14 Servizio di Bioarcheologia, Museo delle Civiltà, Rome, Italy.; 15 Dipartimento dei Beni Culturali, Università di Padova, Padua, Italy.

## Abstract

The remains of those who perished at Herculaneum in 79 CE offer a unique opportunity to examine lifeways across an ancient community who lived and died together. Historical sources often allude to differential access to foodstuffs across Roman society but provide no direct or quantitative information. By determining the stable isotope values of amino acids from bone collagen and deploying Bayesian models that incorporate knowledge of protein synthesis, we were able to reconstruct the diets of 17 adults from Herculaneum with unprecedented resolution. Significant differences in the proportions of marine and terrestrial foods consumed were observed between males and females, implying that access to food was differentiated according to gender. The approach also provided dietary data of sufficient precision for comparison with assessments of food supply to modern populations, opening up the possibility of benchmarking ancient diets against contemporary settings where the consequences for health are better understood.

## Teaser

Compound-specific isotope analysis of human bone collagen provides a snapshot of dietary variability at Roman Herculaneum.

## INTRODUCTION

**Fig. 1:**
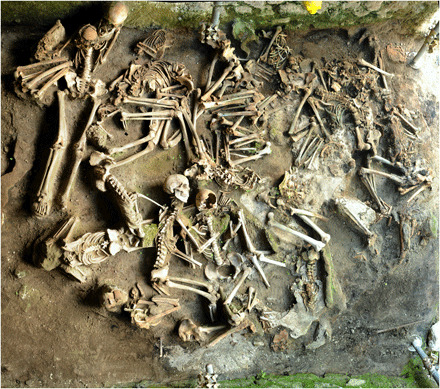
View of skeletal remains in one of the vaulted chambers (fornici) during excavation. Photo credit: L. Fattore, Sapienza Università di Roma.

The human remains found at Herculaneum represent a sample of a “living” population who died trying to escape from the eruption of the Vesuvius volcano in 79 CE. In total, 340 individuals have been excavated from the beach and from nine adjacent fornici (stone vaults) that run parallel to the seashore, where they sought shelter (Fig. 1) (*1*). This remarkable assemblage of victims of a natural catastrophe not only is of huge public interest but also offers an opportunity to substantially advance our knowledge of Roman society through the application of bioarchaeological approaches. The skeletal sample at Herculaneum is not constrained by the biases usually faced by osteoarchaeologists when dealing with attritional cemetery assemblages, such as selective mortality and burial; rather, it provides a “snapshot” of an ancient population rarely afforded in archaeology. Although some selectivity between the few who failed to evacuate the town of ca. 3000 to 4000 inhabitants and the majority who escaped may be expected, males, females, the old, and young are all well represented (*1*, *2*). No evidence has emerged as yet of biases toward any particular social class, although we know from other evidence, namely, the so-called Album of Herculaneum, that freedmen and slaves made up a high proportion of the residents of the town (*3*, *4*).

Here, we sought to reconstruct the diets of 17 individuals from this catastrophic death assemblage through compound-specific stable isotope analysis (CSIA) of amino acids (AAs) directly obtained from bone collagen. The aim of this study was to quantify and examine dietary variability within this unique sample of Roman society at much higher resolution than has previously been achievable (*5*–*7*), particularly by deploying a Bayesian model that incorporates prior knowledge of AA metabolism. We examine the capacity of this method to provide nutritional information regarding major food classes that were available and compare their consumption across the sample. In doing so, we hoped to create an approach for dietary reconstruction that would be suitable for much wider application.

Despite its importance for assessing health and well-being, quantitative data regarding food supply and diet are rarely available to historians, leaving only impressionistic accounts of consumption. Literature, epigraphy, and other documentary evidence, including papyri, can be a useful source of information for social and economic historians, but they are often anecdotal, difficult to quantify, and far from complete, and even the most detailed accounts of consumption practices usually only refer to a narrow stratum of society (*2*). Faunal and botanical remains recovered from archaeological excavations provide detailed evidence of the range of the foods available, and quantitative analysis can reveal major economic changes through time (*8*, *9*), but both are subject to sample and taphonomic biases and only rarely they can be reconciled with specific household activities [e.g., (*10*)], let alone individual diets. These gaps in our knowledge limit our ability to meaningfully compare diets either through time or by geographical location. In addition, we have only limited knowledge of how diets may have varied within an ancient society, for example, by social standing, gender, or between households, villages, or towns or over the course of an individual’s life. Without accurate quantification, we are unable to make fruitful comparisons among ancient populations or with modern societies, where more robust and detailed nutritional data are available. Such comparisons are essential for studying the long-term relationship between diet, health, disease, environmental change, and social inequality and the origin and changing nature of food cultures.

Following its first application over four decades ago (*11*), stable isotope analysis (SIA) of bone collagen offered a way to circumvent these problems by providing dietary estimates that can be compared across time and space. The approach has penetrated all aspects of archaeology and anthropology, offering dietary information regarding specific individuals, from Neanderthals to historical figures (*12*, *13*), and insight into differential access to foodstuffs within populations (*14*). The carbon and nitrogen in adult bone collagen derive from foods typically consumed over a period of at least 10 years before death (*15*), and their respective collagen isotope ratios, expressed as δ^13^C and δ^15^N values, are related to those in the foodstuffs consumed over this period. Atoms in collagen are derived from AAs either incorporated directly from dietary proteins (source AAs) or synthesized de novo (trophic AAs), the latter using additional carbon from proteins, carbohydrates, and lipids (*16*), and nitrogen from transamination reactions with the metabolic pool of amino nitrogen (*17*). This integrated bulk isotopic signal is immensely powerful at providing long-term dietary records, but the approach relies on knowledge of the proportion of AAs routed to collagen directly from the diet against those synthesized de novo by the body. While source AAs undergo negligible isotopic fractionation, trophic AAs are synthesized by a series of transamination and deamination reactions, leading to significant isotopic fractionations (*18*). Understanding the magnitude of these isotopic changes under different dietary scenarios is a major challenge still outstanding in this field, severely limiting the accuracy of the approach.

A range of controlled studies and feeding experiments have been undertaken to understand both the degree of fractionation and the extent of AA routing. It has more recently emerged that the latter is likely to be itself dependent on dietary composition (*19*), further reducing the reliability of dietary reconstructions based on bulk carbon and nitrogen isotopic values. Moreover, the degree of fractionation between food and consumer tissues has also been found to be variable in animal feeding experiments and controlled dietary studies of humans (*20*). To overcome these sources of uncertainty, isotope ecologists and archaeological scientists are turning to measurements of the isotopic signatures of individual AAs (*18*, *21*–*23*), which can be more easily traced to specific dietary sources. Such CSIA approaches are beginning to reveal additional dietary information that is often obscured in bulk stable isotope datasets, allowing population-level dietary patterns to be tracked through time and space at much greater resolution (*22*). However, here, we focus on the utility of CSIA to explore intrapopulation dietary differences. Rather than using AA isotope proxies to distinguish dietary groups, we use previous knowledge of the AA metabolic pathways, their dietary isotope values, and their dietary concentrations to quantify individual diets using probabilistic models (*24*). We aimed to examine whether the differences between individuals at Herculaneum, as shown from bulk SIA (*7*), could be refined and quantified at higher precision.

## RESULTS

We extracted collagen and measured the δ^13^C and δ^15^N values of AAs by gas chromatography–combustion–isotope ratio mass spectrometry (GC-C-IRMS) from the ribs and one tarsal bone (individual F10i22) of 11 adult males and 6 adult females whose remains were found within the vaulted chambers (fornici) next to the Herculaneum beachfront (Materials and Methods and table S1). We considered three potential food groups (C_3_ cereals, terrestrial animals, and marine fish) as the most likely dietary sources for people living in 79 CE Herculaneum, based on archaeological finds from the site (*10*) and historical records (*25*). We obtained baseline δ^13^C_AA_ and δ^15^N_AA_ values from the collagen of terrestrial animals (omnivores and herbivores) and marine fish bones, the majority from first century CE contexts at Herculaneum and Pompeii (table S2). As endogenous AAs cannot be reliably extracted from archaeological plant remains, which are often charred, an alternative strategy was used. Bulk and AA stable isotope values were first measured in modern grains to derive an offset for each AA. AA stable isotope values of archaeological cereal grains were then predicted by applying the offsets to bulk measurements of cereal grains from Herculaneum and previously reported values from comparable Roman contexts (Materials and Methods and table S3) (*26*, *27*). Last, Bayesian mixing models were applied to explore the data considering uncertainties in the isotope measurements and the concentration of AAs and macronutrients in the different potential foodstuffs.

**Fig. 2:**
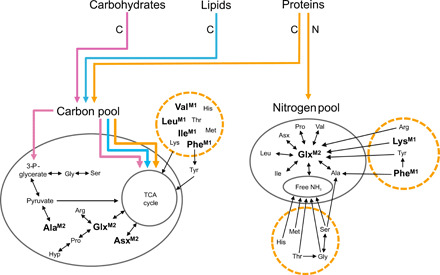
Rationale for metabolic model parameters. Carbohydrates, lipids, and proteins all contribute to the “metabolic carbon pool”: carbon in alanine, serine, and glycine has a glycolytic origin, which is directly linked to carbohydrate digestion; glutamic acid and aspartic acid are synthesized via transamination through the TCA cycle from all macronutrients (*33*). Dietary protein is considered to be the only source of nitrogen, with glutamic acid as the source of nitrogen for other trophic AAs (*17*). “Source” AAs incorporated directly from diet with negligible isotopic fractionation are indicated by dashed circles. Isotope values for AAs labeled M1 and M2 are used in model 1 and model 2, respectively. Ala, alanine; Gly, glycine; Val, valine; Leu, leucine; Ile, isoleucine; Thr, threonine; Ser, serine; Pro, proline; Asx, aspartic acid/asparagine; Glx, glutamic acid/glutamine; Phe, phenylalanine; Lys, lysine; Tyr, tyrosine; His, histidine; Arg, arginine.

**Fig. 3:**
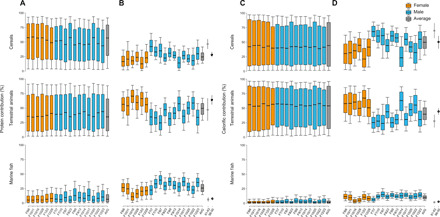
Dietary estimates for 17 individuals from Herculaneum under different scenarios. Estimates were obtained using a concentration-dependent Bayesian mixing model. (**A**) Model 0_p_: SIA, protein routed model. (**B**) Model 1: CSIA, protein model. (**C**) Model 0_wd_: SIA, whole diet model. (**D**) Model 2: CSIA, whole diet model. Boxes represent a 68% credible interval (corresponding to the 16th and 84th percentiles), while the whiskers represent a 95% credible interval (corresponding to the 2.5th and 97.5th percentiles). The horizontal continuous line represents the estimated median (50th percentile). Orange, females; blue, males; gray, outcomes based on average AA isotopic values of the 17 individuals. Equivalent proportions of protein and calorie supplied to modern Mediterranean populations between 1961 and 1963 (gray circles) and 1998 and 2000 (black circles) are shown, with bars representing 1 SD (*28*).

In model 1, we considered only nitrogen and carbon isotope values of source AAs [leucine (Leu), valine (Val), isoleucine (Ile), and phenylalanine (Phe) for δ^13^C and Phe and lysine (Lys) for δ^15^N] that we were able to reliably measure in ancient bone collagen (fig. S1) and modern cereals (fig. S2). As these AAs show negligible (<1‰) isotopic fractionation between diet and consumer and are derived only from dietary protein (Fig. 2), they offer the most robust approach for estimating the composition of ancient human diets because the major assumptions regarding fractionation and routing are negated. Using this approach, we were able to easily discriminate the three different food groups at Herculaneum, implying fundamental isotopic differences in the AAs of primary producers in their respective food sources (i.e., cereals, animal forage, and marine phytoplankton). The estimates obtained from model 1 (Fig. 3B) represent % component contribution to total dietary protein (by dry weight). Using this approach, we achieve far higher dietary resolution compared to previous approaches that rely on bulk collagen stable isotope data alone (Fig. 3A) (*5*), with individual estimates of each food group typically ±10% at the 68% credible interval. We show that the bulk isotope data underestimate the marine protein component of diet, leading to an erroneous interpretation of the importance of fish to the inhabitants of this coastal town. When the AA data are considered, the marine contribution is shown to be nonnegligible (mean = 26 ± 6%) for all individuals, in line with estimations based on ^14^C marine reservoir ages (*6*) and supported by other assessments of the economy of the Bay of Naples during the first century CE (*10*). The estimated marine protein consumption at Herculaneum is notably higher than the relative amounts of marine protein supplied to mid- and late-20th century Mediterranean populations (*28*), which are consistently below 10% (Fig. 3).

**Fig. 4:**
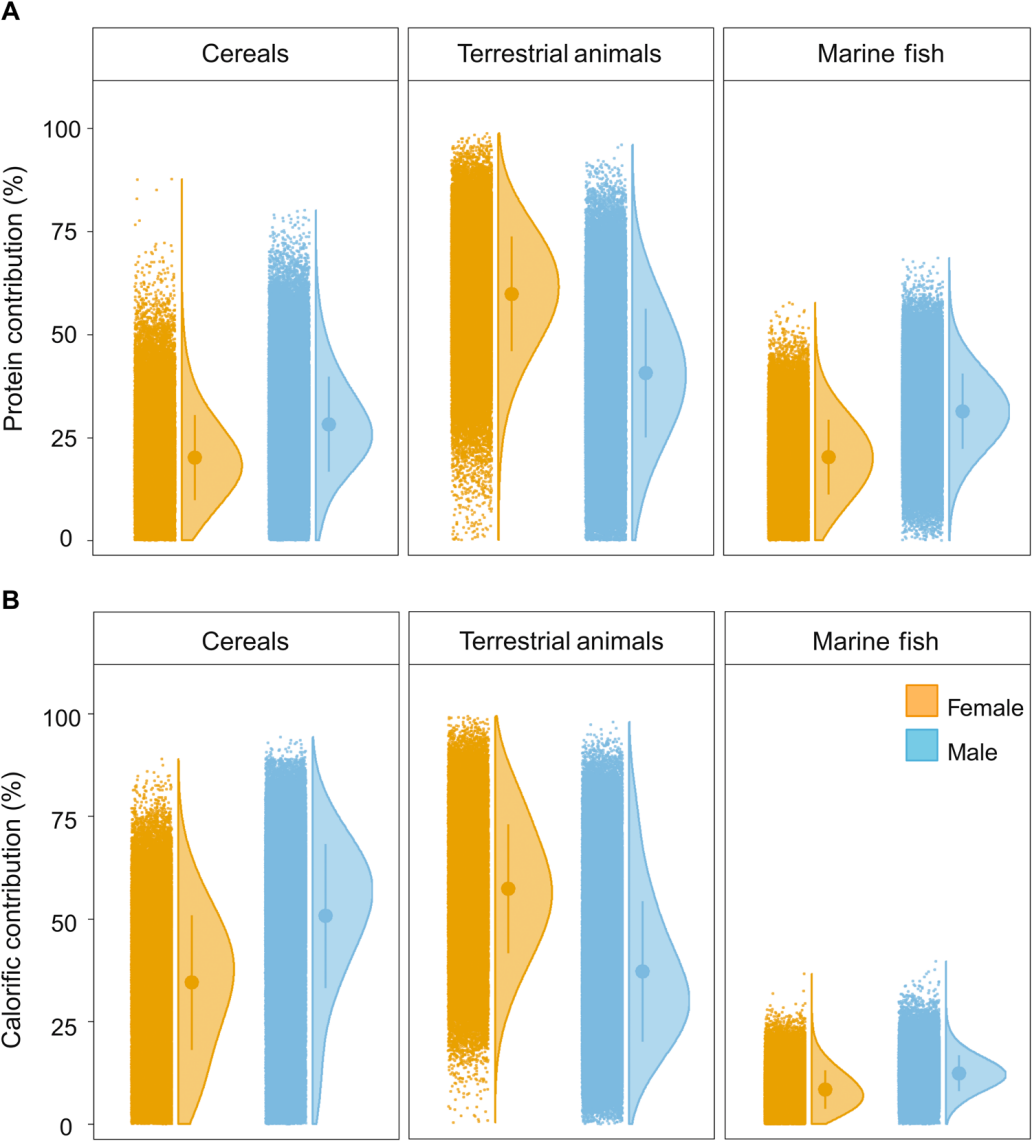
“Raincloud” plots of dietary estimates for 17 individuals from Herculaneum grouped by sex. Estimates were obtained using a concentration-dependent Bayesian mixing model. (**A**) Model 1: CSIA, protein model. (**B**) Model 2: CSIA, whole diet model. The rainclouds show the raw outputs of each model alongside the means and SDs and the probability density of the distribution. Nonparametric Wilcoxon test (two-sided) shows statistical differences for all the food sources across sex when applied to both model 1 and model 2 (*P* < 0.05; table S5).

The source AAs also show significant sex-based dietary differences throughout the group for all food sources (Fig. 4 and table S5), with females generally obtaining less of their total dietary protein from fish and cereals than males but relatively more from terrestrial animal products (i.e., meat, eggs, and dairy). This last category could also theoretically include protein from a broad range of locally produced foods, including pulses, legumes, and nuts, as these foodstuffs are likely to have had similar isotope values of source AAs to animal forage. It has previously been demonstrated from bulk isotope datasets that males had greater access to marine fish at Herculaneum (*7*) and more broadly in Roman Italy (*29*, *30*). Males were more likely to be directly engaged in fishing and maritime activities; they generally occupied more privileged positions in society and were freed from slavery at an earlier age, providing greater access to expensive commodities, such as fresh fish (*7*, *31*). However, here, we were able to quantify the gender gap more accurately within the group, with males, on average, obtaining 1.6 times more dietary protein from seafood compared with females (Fig. 4A). Males also obtained a higher proportion of protein from cereals compared with their female contemporaries, whereas females obtained a greater proportion of protein from terrestrial animal products or locally grown plant foods. Although these estimates do not reflect the absolute quantities of protein consumed, which also may have varied considerably by gender, such a quantitative approach is likely to be immensely useful for studying nutritional health in ancient societies, especially when used in conjunction with historical sources.

Next, we estimated the contribution of each source to the total diet by dry weight, broadly equivalent to the contribution to total calorific value. To do so, we considered the additional contribution of carbon from dietary carbohydrates and lipids. We adapted the concept of “metabolic pools” (*17*) from which carbon and nitrogen are drawn for AA synthesis. This model (model 2) additionally considers trophic AAs: alanine (Ala), glutamine/glutamic acid (Glx), and asparagine/aspartic acid (Asx) as sources of carbon. The carbon in Ala is considered to have a glycolytic origin and therefore to have been obtained from the digestion of carbohydrates via pyruvate (Fig. 2). Conversely, the carbon in Glx and Asx is derived from intermediates of the tricarboxylic acid cycle (TCA) and therefore considered to have been derived from the pool of carbon from all macronutrients including protein (Fig. 2). These proxies are confirmed by the high correlations observed in δ^13^C values between AAs and dietary macronutrients from controlled feeding experiments (*16*, *32*–*34*). The δ^13^C values of dietary protein, carbohydrates, and lipids are estimated from the bulk δ^13^C values of faunal collagen or plant remains using previously established macronutrient “offsets” updated after more recent studies (Supplementary Materials and Methods) (*19*, *35*–*37*). Last, model 2 also considered Glx as an additional source of nitrogen. The difference in δ^15^N of Glx and Phe has been used to study an organism’s trophic position (*18*), but alone, they fail to resolve more complex diets, as in this case, when there are multiple sources (fig. S3). As glutamic acid is involved in transamination of other AAs, its nitrogen is considered to derive from the total pool of nitrogen and therefore is estimated from the bulk δ^15^N value of each protein source (Fig. 2) (*17*). The estimation of nitrogen isotopic fractionation associated with interchange of nitrogen between glutamic acid/glutamine and the nitrogen pool was obtained from studies of a range of consumers and their food sources (Supplementary Materials and Methods).

Compared to model 1, model 2 introduces additional sources of uncertainty regarding the degree of trophic AA fractionation, energy macronutrient source values, and the flux of both carbon and nitrogen from dietary pools to collagen AAs. Nevertheless, even by using conservative estimations of these errors (Supplementary Materials and Methods and data file S1), the output of model 2 shows much greater dietary resolution compared to using bulk data alone (Fig. 3C) (*5*), with a nonnegligible contribution of marine foods to total calories for the majority of individuals and a statistical difference between sexes for all foodstuffs (Fig. 3 and table S5). The estimations of calorific value provided by model 2 also correspond well with previous estimations of % marine carbon in diet based on their marine reservoir ages (fig. S4) (*6*, *7*). The % dietary protein contribution estimated from model 2 is also within the error of those from model 1, providing further cross-validation (table S4).

The results of model 2 show that, on average, individuals at Herculaneum obtained the majority of their energy from terrestrial resources, i.e., cereals (49 ± 10%) and terrestrial animal products (40 ± 10%). However, other high-energy products such as olive oil, and potentially wine, are not considered as dietary sources and therefore missing in the outputs provided in Fig. 3. Olive oil, for example, contributes ca. 5% of the calories in contemporary Mediterranean populations (*28*). By using the δ^13^C value of modern Mediterranean olive oils (*38*–*40*) corrected for the Suess effect (*41*), model 2 permits a contribution of 29 ± 17% to total diet, when it is included as an additional source (Supplementary Materials and Methods and data file S1). Although even the lowest estimation would be much higher than most modern Mediterranean populations, this value is consistent with estimations of oil consumption in Rome during the first century CE [ca. 20 liters/year (*42*)], directly attesting to the importance of the olive as one of the triads of the Roman Mediterranean diet, along with cereals and wine (*25*).

## DISCUSSION

By applying the CSIA approach to the Herculaneum sample, here, we are able to reconstruct the diets of people who lived contemporaneously with unprecedented resolution compared to previous studies (*5*–*7*). We show with much greater certainty that adult males and females drawn from the sample population had different diets during their lifetime. This must be attributable to differential access to foodstuffs, perhaps related to the different occupations held by men and women, cultural prohibitions, or evidence of the uneven distribution of power that restricted certain foods to the latter (*25*). A clear distinction by sex, however, is not observed in all cases. The dietary estimates from the male sample were more variable than the female, with some males consuming less cereal-based foods than the others (Fig. 3, B and D), perhaps related to differences in their occupation or social standing, aspects difficult to directly assess given the nature of the assemblage. It is significant that such subtle dietary differences are not observable from the lower-resolution reconstructions based on the bulk isotope data alone (Fig. 3, A and B).

The paleodietary data obtained from CSIA are also of sufficient quality for comparison with records of food supplied to modern populations. We found that proportionally more marine foods were consumed by the inhabitants of first century Herculaneum compared to 20th century Mediterranean populations, while cereals were of lower overall dietary significance compared to the typical “Mediterranean diet,” as defined in the 1960s (*28*). Whether this pattern is reflected more broadly in ancient Mediterranean societies or is peculiar to coastal settlements, such as Herculaneum, remains to be determined. Such high-resolution data also open up the possibility of “benchmarking” ancient diets against modern records, where, for example, the nutritional consequences for health are better understood [e.g., (*43*)].

More broadly, we show that CSIA of collagen AAs combined with probabilistic modeling, as presented above, offers a robust approach for dietary reconstruction at unprecedented resolution. This is an important advance that is likely to transform paleodietary research, not least by providing data that are of adequate quality to be of interest to the broader community of nutritional and environmental scientists. For example, quantification of seafood consumption by past communities could be used to study long-term anthropogenic impacts on marine ecosystems (*44*) or help assess health inequalities (*45*). Dietary accuracy is greatly enhanced by our knowledge of the δ^13^C_AA_ and δ^15^N_AA_ values of the main food groups under consideration, and so, obtaining these data from a broader range of non-osseous sources, such as legumes, nuts, fungi, and wild plant foods, would be a fruitful focus for future research. Last, we show that using bulk stable isotope data alone to reconstruct an individual’s diet can lead to erroneous conclusions regarding the relative quantities of different foodstuffs consumed and the extent of dietary variability within ancient populations.

## MATERIALS AND METHODS

### Experimental design

The ribs and one tarsal bone (individual F10i22) of 17 adult individuals were obtained from vaulted chambers (fornici) next to the Herculaneum beachfront. Nine had previously been subjected to radiocarbon dating, and all had had a full osteological assessment (table S1). Further samples that previously yielded the highest amounts of collagen (*1*) were preferentially selected for analysis. Collagen was extracted and analyzed by elemental analysis IRMS (EA-IRMS) and prepared for GC-C-IRMS following hydrolysis to release AAs. The same procedure was applied to faunal remains from the study area (table S2). Stable carbon and nitrogen isotope measurements were obtained for at least nine individual AAs for each extract. Procedures were made for assuring quality control (Supplementary Materials and Methods). AA stable isotope values were also obtained from modern cereals, and these data were used to estimate the values for ancient cereals based on their bulk isotope values (table S3). Several mixing models were constructed using the knowledge of the bulk and AA stable isotope values in the source foodstuffs, the concentrations of AAs in the foodstuffs, and their associated uncertainties (Supplementary Materials and Methods). The outputs of the models were used to create Figs. 3 and 4 and derive inferences.

### Collagen extraction

Collagen was extracted from bone fragments following the modified Longin method (*46*). Briefly, small human and animal bone fragments (ca. 100 to 500 mg) were mechanically cleaned to remove exogenous residues and demineralized at +4°C in 8 ml of 0.6 M HCl for at least 48 hours. A homogenized modern bovine bone sample was included with each batch of sample to serve as a control. More fragile fish elements were demineralized with a more diluted HCl solution (0.1 M). Once completely demineralized, collagen was gelatinized at 80°C for 48 hours in 0.001 M HCl. Gelatinized collagen was filtered (60 to 90 μm; Ezee filters), ultrafiltered (Amicon Ultra-4 Millipore 30 kDa of Ultracel membrane), and then freeze-dried.

### Elemental analysis isotope ratio mass spectrometry

Collagen (0.9 to 1.1 mg) was analyzed in duplicate using a Sercon continuous flow 20-22 IRMS interfaced with a Universal Sercon gas solid liquid elemental analyzer to determine the carbon and nitrogen isotopic values. The obtained values were corrected from the isotopic ratio of the international standards, Vienna Pee Dee Belemnite (VPDB) for carbon and air (AIR) for nitrogen, using the standard δ (‰) notation.

Uncertainties on the measurements were calculated by combining the SDs of the sample replicates and those of reference material according to Kragten (*47*). Caffeine (IAEA-600), ammonium sulfate (IAEA-N-2), and cane sugar (IA-Cane) international standards were used as reference material in each analytical run. International standard average values and SD across the runs were as follows: IAEA-600 (*n* = 25), δ^13^C raw = −27.69 ± 0.15‰ (δ^13^C true = −27.77 ± 0.04‰) and δ^15^N raw = +0.99 ± 0.26‰ (δ^15^N true = 1 ± 0.2‰); IAEA-N-2 (*n* = 25), δ^15^N raw = +20.32 ± 0.15‰ (δ^15^N true = 20.3 ± 0.2‰); and IA-CANE (*n* = 24), δ^13^C raw = −11.67 ± 0.11‰ (δ^13^C true = −11.64 ± 0.03‰). The maximum uncertainty across all samples (*n* = 83) was ±0.41‰ for δ^13^C and 0.32‰ for δ^15^N.

### Preparation of AAs for GC-C-IRMS

Collagen was hydrolyzed (6 M HCl, 200 μl, 110°C, 24 hours) after addition of 250 μl of an internal norleucine standard (Sigma-Aldrich) of known isotopic composition. The hydrolysates were centrifuged (11,000*g*, 1 min) using Pall Nanosep filters (0.45 μm) to remove the remaining insoluble material. The hydrolysates were gently dried at room temperature under N_2_, redissolved in 0.1 M HCl (100 μl), and stored at −20°C until required for analysis. Samples were again evaporated to dryness before derivatization. Amino acids were then derivatized to form *N*-acetyl-*i*-propyl (NAIP) esters (*48*).

Briefly, isopropanol and acetyl chloride (1 ml; 4:1 v/v) were added, and tubes were sealed and heated at 100°C (1 hour). After 1 hour, sample mixtures were cooled (at −20°C), and the solution was dried under a gentle stream of N_2_. Dichloromethane (DCM) was added (2 × 0.5 ml) and blown down under a gentle stream of N_2_ to remove excess reagents. Next, a mixture of acetic anhydride, triethylamine, and acetone (1 ml; 1:2:5, v/v/v) was added to the tubes and heated at 60°C (10 min). The mixture was cooled and evaporated to dryness under a gentle stream of N_2_. NAIP esters were then dissolved in ethyl acetate (EtAc; 2 ml), and a saturated NaCl solution (1 ml) was added to separate polar and/or inorganic components from the organic phase and transferred into a new culture tube. The phase separation was repeated with additional EtAc (1 ml). Trace water was removed from the organic phase with molecular sieves (sodium aluminum silicate, 0.3 nm; Merck KGaA, Darmstadt, Germany). The EtAc containing the NAIP esters was blown down under a gentle stream of N_2_, and then DCM (1 ml) was added and dried to remove excess water. Samples were redissolved in known quantities of EtAc and stored at −20°C until required for analysis by GC-C-IRMS. The same derivatization procedure was used for preparing mixtures of international reference standards (Indiana, USA and SHOKO Science, Japan) and standards purchased from Sigma-Aldrich (Sigma-Aldrich Company Ltd., UK).

### Gas chromatography–combustion–isotope ratio mass spectrometry

GC-C-IRMS measurements of the AAs were conducted using a Delta V Plus IRMS (Thermo Fisher Scientific, Bremen, Germany) linked to a Trace Ultra gas chromatograph (Thermo Fisher Scientific, Bremen, Germany) with a GC IsoLink II interface fitted with a Cu/Ni combustion reactor maintained at 1000°C. Ultrahigh-purity–grade helium with a flow rate of 1.4 ml min^−1^ was used as the carrier gas, and parallel acquisition of flame ionization data was achieved by diverting a small part of the flow to an integrated flame ionization detector (Thermo Fisher Scientific). Ethyl acetate was used to dilute the samples, and 1 μl of each sample and 2 μl of each standard were injected at 240°C with a 3.5-spre-injection dwell time onto a custom DB-35 fused silica column (60 m × 0.32 mm × 0.50 μm; Agilent J&W Scientific Technologies, Folsom, CA, USA). All samples were injected in triplicate. The oven temperature program used for samples and standards was as follows: 40°C (hold 5 min) and then increasing by 15°C min^−1^ up to 120°C, then by 3°C min^−1^ up to 180°C, then by 1.5°C min^−1^ up to 210°C, then by 5°C min^−1^ up to 280°C (hold 8 min).

A Nafion membrane removed water, and a cryogenic trap was used to remove CO_2_ from the oxidized and reduced sample when operated in nitrogen mode. In carbon mode, eluted products were combusted to CO_2_ and ionized in a mass spectrometer by electron impact. Ion intensities of mass/charge ratio (*m/z*) 44, 45, and 46 were monitored to automatically compute the ^13^C/^12^C ratio of each peak in the samples. In nitrogen mode, ion intensities of *m/z* 28, 29, and 30 were monitored to automatically compute the ^15^N/^14^N ratio of each peak in the samples. Computations were made with Isodat (version 3.0; Thermo Fisher Scientific) and were based on comparisons with a repeatedly measured high-purity standard reference gas (CO_2_ or N_2_). The results from the analysis are reported in parts per mil (‰) relative to international standards using the δ notation.

### δ^15^N measurements of AAs

Each reported value is a mean of triplicate δ^15^N measurements. An AA international standard mixture of known isotopic composition was run after every three sample injections to monitor instrument performance and drift. The AA standard mixture used for δ^15^N determinations comprised eight international standards (Indiana and SHOKO Science) and l-norleucine (Sigma-Aldrich). δ^15^N true values of l-norleucine were determined in-house by EA-IRMS. International standard average raw values and SD (*n* = 124) across the runs were as follows: Ala, 42.22 ± 3.07‰ (true: +43.25 ± 0.07‰); Gly, +1.09 ± 2.02‰ (true: +1.76 ± 0.06‰); Val, −4.07 ± 1.73‰ (true: −5.21 ± 0.05‰); Leu, +6.39 ± 1.27‰ (true: +6.22‰); Nle, +14.46 ± 1.42‰ (true: +14.31 ± 0.23‰); Asp, +33 ± 1.50‰ (true: 35.2‰); Glu, −3.52 ± 1.10‰ (true: −4.52 ± 0.06‰); Hyp, −8.19 ± 1.08‰ (true: −9.17‰); Phe, +1.73 ± 0.69‰ (true: +1.70 ± 0.06‰). Sample δ^15^N raw values were corrected by the calibration curve and the l-norleucine internal standard true value.

### δ^13^C measurements of AAs

Each reported sample value is a mean of triplicate δ^13^C measurements. Amino acids in the samples were first corrected for the isotopic difference between l-norleucine in the standard mixture and l-norleucine in the sample. δ^13^C AA measurements were then corrected by specific correction factors to account for the derivatizing carbon and the kinetic isotope effect (*49*), according to the following equation

$$\delta^{13}C_{\text{CORR}}=\delta^{13}C_{D}=\frac{\left(n_{\text{DC}}\;\delta^{13}C_{\text{DC}})-(n_{C}\;\delta^{13}C_{C}\right)}{n_{D}}$$

where *n* is the number of carbon atoms, DC indicates the derivatized compound, C is the original compound, and D is the derivative group.

A standard AA mixture was run after every three sample injections, and the average correction factors from the standard mixture were used for the correction of the samples (Sigma-Aldrich, UK). True δ^13^C values of standards were measured by EA-IRMS: Ala, −19.31 ± 0.02‰; Gly, −33.31 ± 0.02‰; Val, −10.89 ± 0.02‰; Leu, −13.78 ± 0.06‰; Ile, −24.89 ± 0.07‰; Nle, −27.59 ± 0.02‰; Thr, −10.46 ± 0.01‰; Ser, −12.54 ± 0.09‰; Pro, −12.33 ± 0.02‰; Asp, −27.52 ± 0.12‰; Met, −29.88 ± 0.14‰; Glu, −28.57 ± 0.09‰; Hyp, −12.52 ± 0.03‰; Phe, −11.52 ± 0.05‰; Lys, −13.7 ± 0.11‰; Tyr, −24.85 ± 0.02‰.

The standard δ^13^C average correction factor values and SD (*n* = 154) across the runs were as follows: Ala, −40.46 ± 1.22‰; Gly, −39.73 ± 1.02‰; Val, −45.57 ± 1.39‰; Leu, −45.03 ± 2.10‰; Ile, −46.31 ± 1.81‰; Nle, −43.43 ± 1.63‰; Thr, −48.52 ± 1.25‰; Ser, −46.56 ± 1.19‰; Pro, −42.27 ± 1.41‰; Asp, −37.27 ± 1.09‰; Met, −41.82 ± 2.12‰; Glu, −36.73 ± 1.10‰; Hyp, −47.97 ± 1.13‰; Phe, −45.36 ± 1.46‰; Lys, −48.29 ± 2.29‰; Tyr, −48.71 ± 1.23‰. Correction factors induce a new source of error; therefore, the error propagated for each AA was calculated according to the following equation (*49*)

$$\sigma^{2}={\sigma^{2}}_{s}\;{\left(\frac{n_{S}}{n_{C}}\right)}^{2}+{\sigma^{2}}_{\text{DS}}(\frac{n_{S}+n_{D}}{n_{C}})+{\sigma^{2}}_{\text{DC}}(\frac{n_{C}+n_{D}}{n_{C}})$$

where σ is the SD, *n* is the number of carbon atoms, S represents the nonderivatized standard, DS is the derivatized standard, DC is the derivatized compound, C is the original compound, and D is the derivative group.

### Analysis of modern and archaeological cereals

Modern C_3_ cereals were collected from Italian organic productions. Three species were selected for the analysis: barley (*Hordeum vulgare*), einkorn wheat or farro (*Triticum monococcum*), and durum wheat (*Triticum durum*). Grains were homogeneously powdered, washed three times with deionized water, and freeze-dried. Around 2 mg was weighed out in duplicate and analyzed by EA-IRMS to measure bulk carbon and nitrogen isotopic values following the approach described above. A portion of the original powdered material was prepared for compound-specific analysis following a slightly modified protocol from Styring *et al*. (*50*, *51*). Lipids were first extracted from the powdered samples with DCM/methanol (2:1 v/v, 10 ml) by ultrasonication, and the extracts were stored at −20°C until required for analysis. Around 40 mg of dry lipid extracted residues was hydrolyzed (6 M HCl, 2 ml, 110°C, 24 hours). A known quantity of internal standard was added at this stage (norleucine, Sigma-Aldrich). The hydrolyzed samples were centrifuged (11,000*g*, 1 min) twice using Nanosep filters to remove the insoluble matter left. The hydrolyzed samples were blown to dryness under N_2_, redissolved in 0.1 M HCl, and stored at −20°C until required for analysis.

Four charred cereals (ca. 300 mg) from excavations at Herculaneum (table S3) were sampled for EA-IRMS analysis, including *H. vulgare* (archive #1703/76981), *Triticum* sp. (#1703/76981 and #723/76000), and *Triticum dicoccum* (#1895/77175). The samples were treated with 0.5 M HCl for 20 min at room temperature to remove external carbonates and then rinsed three times with deionized water. The samples were then frozen and lyophilized, grounded, and weighed into tin capsules for EA-IRMS analysis of both carbon and nitrogen stable isotopes, as described above.

The offset in the δ^13^C and δ^15^N values of each AA and the bulk value was calculated for each of the three modern C_3_ cereal samples (table S3). All AAs in plants are synthesized de novo by following specific metabolic reactions (*52*). Therefore, we assumed that the degree of fractionation of nitrogen and carbon in C_3_ cereal AAs can be predicted relative to their total nitrogen and carbon. Our Δ^15^N_AA-bulk_ values were observed to be similar to those of barley and bread wheat (only grains) published by Styring *et al*. (*52*) and to those of bread wheat published by Paolini *et al*. (*53*).

Next, we predicted AA δ^13^C and δ^15^N values by applying the measured Δ^15^N_AA-bulk_ offsets to the bulk δ^13^C and δ^15^N values from four samples of C_3_ cereals from 79 CE Herculaneum, a barley sample from 79 CE Pompeii (*27*), and four second century CE cereal samples from the Imperial Roman harbor, Portus Romae (*26*). The bulk values of the archaeological grains were corrected for charring after (*54*). From this, we obtained an average value for each AA with an associated uncertainty derived from propagating all errors from the measurements made on charred archaeological cereal grains and the errors associated with the Δ^15^N_AA-bulk_ offset (table S3).

### Statistical analysis

Bayesian mixing models were performed using FRUITS version 3.0 beta (available at http://sourceforge.net/projects/fruits/). Markov chains were obtained in FRUITS using the Markov chain Monte Carlo method with the BUGS software (https://www.mrc-bsu.cam.ac.uk/software/bugs/). The BUGS software applies the Metropolis-Hastings algorithm and automatically discards the first 5000 iterations of the Markov chains and then additionally runs them for 10000 iterations. Convergence was assessed by examining the trace autocorrelation plots generated. Last, the model outputs (Markov chains) were summarized, plotted, and statistically analyzed in R (version 4.0.3) using ggplot2 and the raincloud plot function (https://github.com/RainCloudPlots/RainCloudPlots) (*55*). Parameters and the rationale for the four models deployed (model 0_p_, model 0_wd_, model 1, and model 2) are described in Supplementary Materials and Methods, and the FRUITS files used to generate the outputs are also provided. A nonparametric two-sided Wilcoxon test was used to test whether distribution of median predicted contributions differed between sexes for each food group at the 0.05 significance level. This test was used because of the low sample of independent observations (17 individuals).

## SUPPLEMENTARY MATERIALS

Supplementary material for this article is available at http://advances.sciencemag.org/cgi/content/full/7/35/eabg5791/DC1

## Appendix

View/request a protocol for this paper from *Bio-protocol*.
